# Mapping and ablation of left atrial macro-reentrant tachycardia with a novel circular pulsed field ablation catheter

**DOI:** 10.1016/j.hrcr.2024.11.005

**Published:** 2024-11-13

**Authors:** Tillman Dahme, Mohamed Karim Sheta, Sebastian Gabriel, Harald Marschang

**Affiliations:** Department of Cardiology, Klinikum Esslingen, Esslingen am Neckar, Germany

**Keywords:** Pulsed field ablation, PFA, Electroporation, Atypical atrial flutter, Left atrial macro-reentrant tachycardia, Atrial fibrillation, Pulmonary vein isolation, 3D-mapping

**Key Teaching Points**

• The VARIPULSE™ catheter (Biosense Webster, Irvine, CA) is a novel 10-polar, circular, size-adjustable, pulsed field ablation catheter with integration in the CARTO 3™ 3-dimensional mapping system (Biosense Webster), designed to perform pulmonary vein isolation to treat atrial fibrillation.
• Despite being designed for pulmonary vein isolation, the VARIPULSE platform is also suitable for mapping of atrial tachyarrhythmia.
• Pulsed field ablation delivered with the VARIPULSE ablation system is suitable for termination of left atrial macro-reentrant tachycardia.

## Introduction

Pulsed field ablation (PFA), sometimes referred to as “irreversible electroporation,” has recently evolved as a novel means of ablation to treat cardiac arrhythmia. In contrast to thermal ablation, mainly radiofrequency ablation or cryoablation, PFA has certain advantages, such as tissue selectivity for cardiac tissue over nerve cells, esophageal tissue, or blood vessels and avoidance of collateral damage due to extracardiac tissue heating. Several different catheters for PFA have been introduced to the market recently. One such catheter is the circular, 10-polar, bidirectional, and size adjustable VARIPULSE™ Catheter (Biosense Webster, Irvine, CA). The catheter is designed for anatomic 3-dimensional (3D) mapping and delivery of PFA to the pulmonary veins’ ostia and antra to achieve pulmonary vein isolation (PVI) in patients with atrial fibrillation (AF). It is fully integrated in the CARTO 3™ 3D-mapping system (Biosense Webster). The catheter’s loop diameter can be adjusted from 25 mm to 35 mm to fit the ostium of each PV for ostial ablations and also to apply antral or circumferential ablations to each PV. It is not a true single-shot device because, per protocol, it requires overlapping ablations at the ostium and the antrum of each PV, thus a minimum of 4 ablations per PV is recommended.^1^ Efficacy and safety of the VARIPULSE platform for the treatment of paroxysmal atrial fibrillation have been demonstrated in the recent inspIRE and admIRE trials.^2^, ^3^, ^4^

## Case report

**Figure 1:**
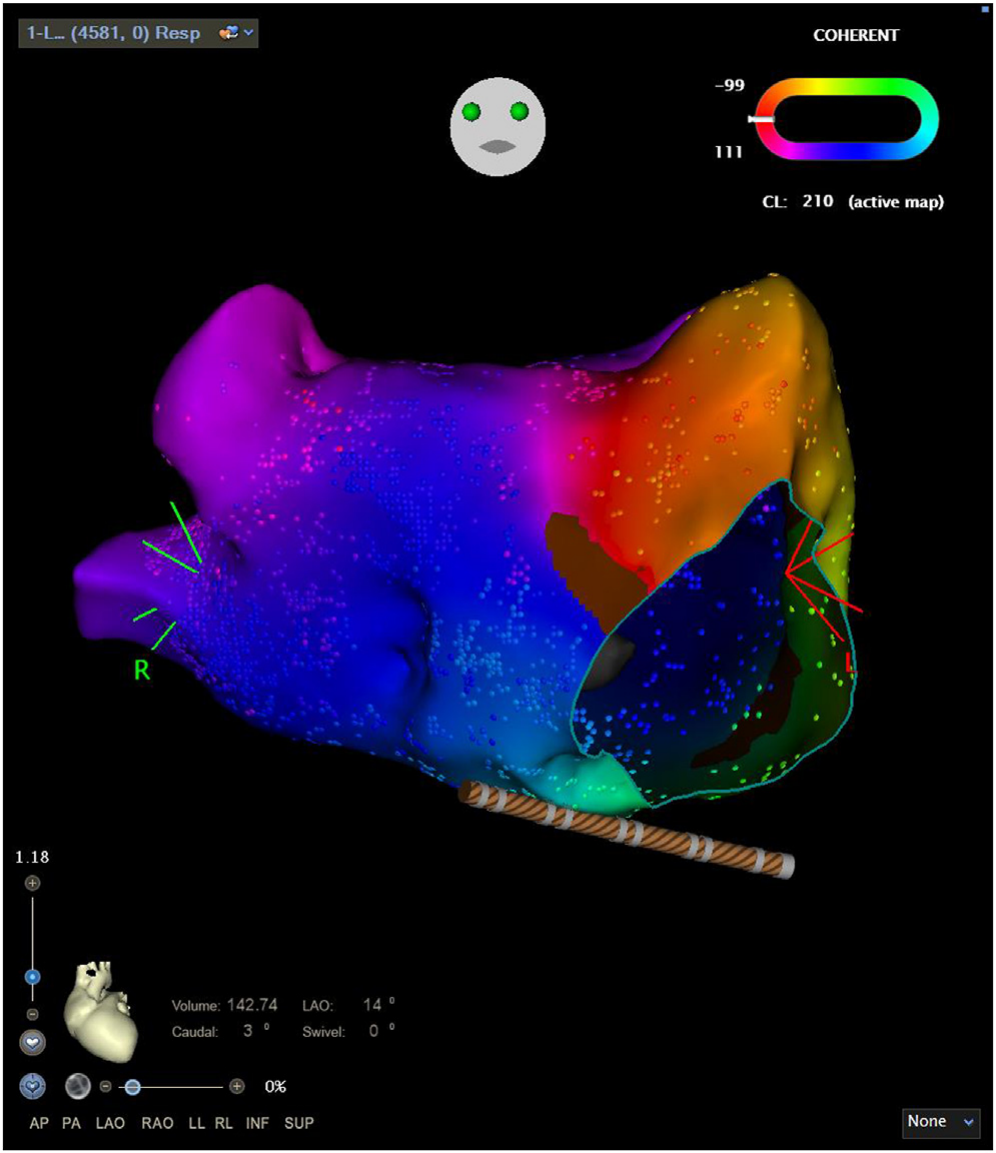
Electroanatomic map of the left atrium obtained with the VARIPULSE catheter (Biosense Webster, Irvine, CA) showing clockwise perimitral flutter.

**Figure 2:**
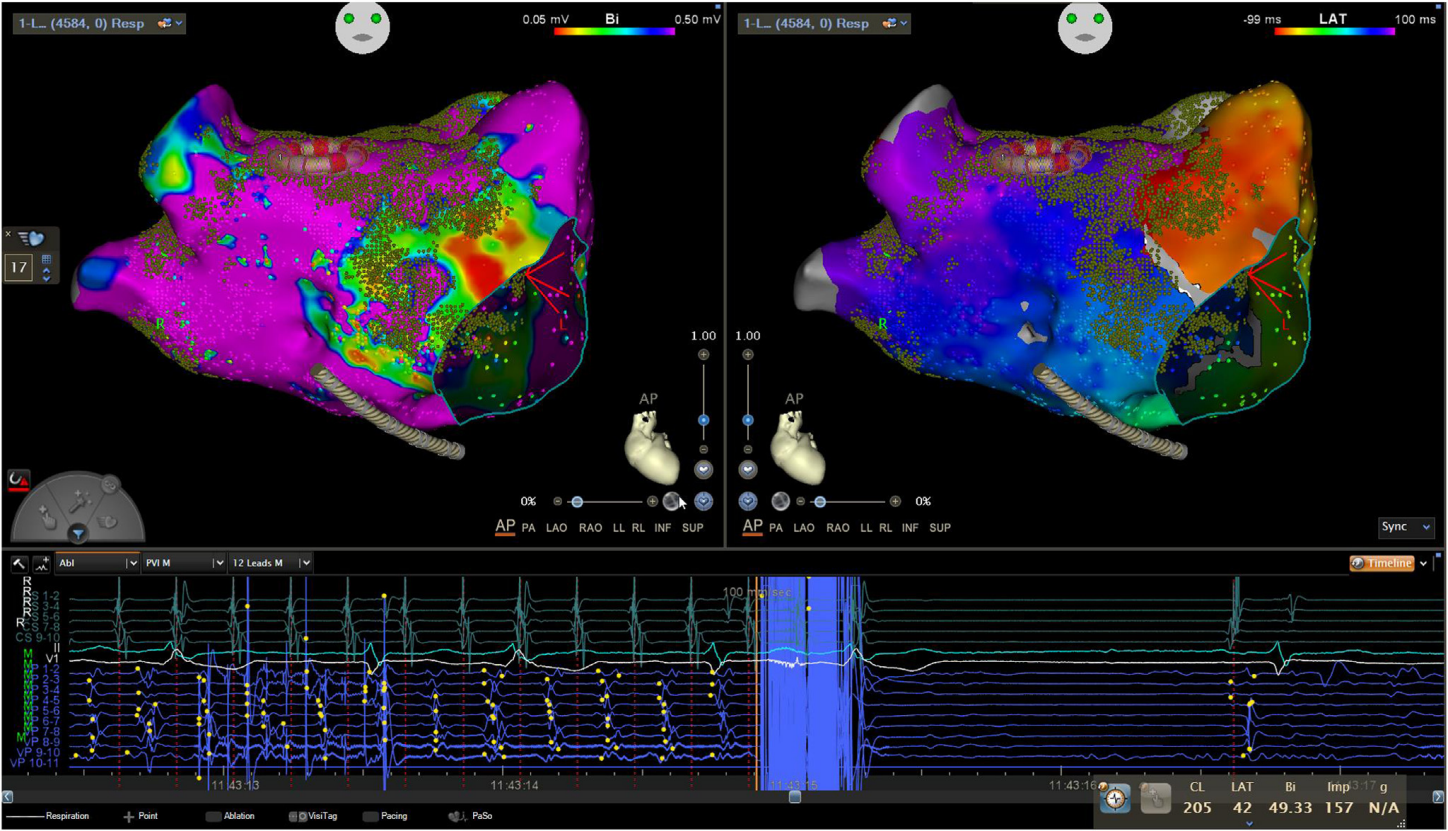
Voltage map (*left upper corner*) and local activation time map (*right upper corner*) of the left atrium and termination of the tachyarrhythmia into sinus rhythm by pulsed field ablation (*bottom*). Ablation sites are indicated by *small green dots* (CARTO VISITAG grid; Biosense Webster, Irvine, CA).

A 60-year-old male patient was scheduled for AF ablation for symptomatic paroxysmal AF. On admission his electrocardiogram showed AF. The patient was scheduled for PVI with the VARIPULSE ablation system. After placement of the coronary sinus (CS) catheter, AF spontaneously organized to a stable narrow-complex tachycardia with an atrial cycle length of 210 milliseconds and eccentric CS activation from the distal to the proximal CS catheter electrodes. Transseptal access was obtained with a steerable sheath (VIZIGO™, Biosense Webster) and a 94-cm transseptal needle (BRK; Abbott, St Paul, MN) under fluoroscopic guidance. Angiography of all PVs was performed to determine the confluence of the PVs into the left atrium (LA). The VARIPULSE catheter was introduced into the LA via the steerable transseptal sheath. 3D electroanatomic mapping (EAM) was initiated. Within 14 minutes of mapping time, an EAM encompassing 4584 electroanatomic points was obtained (Supplemental Video 1). The local activation time and coherent map showed clockwise perimitral macro-reentrant tachycardia and the voltage map showed normal voltage of the LA except for the anterior wall, where substrate could be identified (Figure 1, Supplemental Videos 2 and 3). We then isolated all PVs with PFA with 4 ablations per PV, 2 ostial and 2 antral ablations, which, as expected, did not have any effect on the arrhythmia. We next applied ablations to the anterior wall of the LA with the VARIPULSE catheter in the small loop (25 mm) configuration, creating an anterior line from the left superior PV to the mitral annulus, taking into account the identified substrate.^5^^,^^6^ This led to an increase in the cycle length to 230 milliseconds and a change in the activation pattern on the CS catheter, consistent with roof-dependent atrial flutter. Application of further PFAs to connect the superior PVs (roof line) led to termination of the left atrial macro-reentrant tachycardia into sinus rhythm during ablation (Figure 2 and Supplemental Video 4). No further atrial tachyarrhythmias could be induced by atrial burst stimulation. The patient was discharged on the day after the procedure. No complications occurred. To date, 2 months after the ablation, the patient has not experienced any arrhythmia recurrence.

## Discussion

PFA is an emerging technology to treat atrial arrhythmia and several novel PFA devices and systems have been developed. The VARIPULSE platform is such a novel ablation system, designed for single-shot isolation of PVs to treat AF. The catheter has 10 irrigated electrodes, which have been designed to deliver PFA, but its full integration into the CARTO 3D-mapping system also allows EAM. This can be used to create maps to guide PVI but, in principle, also to map atrial tachyarrhythmia. To the best of our knowledge, this is the first report on the use of the VARIPULSE platform to map and ablate left atrial macro-reentrant tachycardia with this device. Compared with modern high-density, high-resolution mapping catheters, such as the Octaray™ catheter (Biosense Webster) or the Advisor™ HD Grid catheter (Abbott, Abbott Park, IL), the 3-mm-long electrodes are relatively bulky and thus the resolution of maps should be much lower. Nevertheless, we obtained a map comprising more than 4000 electroanatomic points of the left atrium, which certainly may not be able to compete with high-density maps obtained with designated mapping catheters, but was still sufficient to guide ablation of the arrhythmia. The possibility of treating macro-reentrant and most likely focal atrial tachycardia as well, will certainly increase the usefulness of such single-shot, or possibly better termed “quick” PVI devices because they, like the VARIPULSE platform, often need more than a single ablation. This is a major difference from other single-shot systems, such as cryoballoon, which does not provide the possibility to perform EAM without the use of additional mapping catheters and an additional 3D-mapping system.

## Conclusion

The VARIPULSE PFA ablation platform—aside from isolating PVs—is suitable for mapping of left atrial macro-reentrant tachycardia and termination by means of PFA delivered via this catheter is feasible.

## Disclosures

Tillman Dahme has received speaker’s honoraria from Biosense Webster/Johnson & Johnson, Boehringer Ingelheim, Medtronic, AstraZeneca, Bayer, Daiichi Sankyo, Novartis, Sanofi Aventis; the rest of the authors have no conflicts of interest.
