# Supplementary material for: Mapping and ablation of left atrial macro-reentrant tachycardia with a novel circular pulsed field ablation catheter
Source: HeartRhythm Case Rep. 2024 Nov 13;11(2):164–6. doi: 10.1016/j.hrcr.2024.11.005 (PMC11861958; doi:10.1016/j.hrcr.2024.11.005)
Supplement: Supplementary Data [file mmc1.docx]

Supplementary Materials

Supplementary Movies:

Supplementary Movie 1:

Mapping of the left atrium with the VARIPULSE™ catheter

Supplementary Movie 2:

Local activation time (LAT) map (left) and voltage map (right) of the left atrium showing clockwise perimitral flutter and anterior substrate close to the mitral annulus.

Supplementary Movie 3:

LAT propagation map (left) and coherent map (right) documenting clockwise perimitral flutter.

Supplementary Movie 4:

Termination of the macro-reentrant tachycardia by pulsed field ablation.
